# Nutritional Risk Screening 2002 as a Predictor of Postoperative Outcomes in Patients Undergoing Abdominal Surgery: A Systematic Review and Meta-Analysis of Prospective Cohort Studies

**DOI:** 10.1371/journal.pone.0132857

**Published:** 2015-07-14

**Authors:** Zhen Sun, Xin-Juan Kong, Xue Jing, Run-Jun Deng, Zi-Bin Tian

**Affiliations:** Department of Gastroenterology, Affiliated Hospital of Medical College of Qingdao University, Qingdao, 266003, Shandong Province, China; University Hospital Oldenburg, GERMANY

## Abstract

**Background:**

The nutritional risk screening (NRS 2002) has been applied increasingly in patients who underwent abdominal surgery for nutritional risk assessment. However, the usefulness of the NRS 2002 for predicting is controversial. This meta-analysis was to examine whether a preoperative evaluation of nutritional risk by NRS 2002 provided prediction of postoperative outcomes in patients undergoing abdominal surgery.

**Methods:**

A systematic literature search for published papers was conducted using the following online databases: MEDLINE, EMBASE, the Cochrane library, EBSCO, CRD databases, Cinahl, PsycInfo and BIOSIS previews. The pooled odds ratio (OR) or weight mean difference (WMD) was calculated using a random-effect model or a fix-effect model.

**Results:**

Eleven studies with a total of 3527 patients included in this study. Postoperative overall complications were more frequent in nutritional risk patients versus patients without nutritional risk (the pooled OR 3.13 [2.51, 3.90] p<0.00001). The pooled OR of mortality for the nutritional risk group and non-nutritional risk group was 3.61 [1.38, 9.47] (p = 0.009). Furthermore, the postoperative hospital stay was significant longer in the preoperative nutritional risk group than in the nutritional normal group (WMD 5.58 [4.21, 6.95] p<0.00001).

**Conclusions:**

The present study has demonstrated that patients at preoperative nutritional risk have increased complication rates, high mortality and prolonged hospital stay after surgery. However, NRS 2002 needs to be validated in larger samples of patients undergoing abdominal surgery by better reference method.

## Introduction

The reported prevalence of malnutrition in patients scheduled for abdominal surgery is up to 50% [1,2]. Malnutrition is mainly caused by decreased oral intake, tumor-related cachexia, impaired digestive capacity and destruction of gastrointestinal tract in addition to hypermetabolic, catabolic state created by stress of surgery [3]. Moreover, it is demonstrated that nutritional risk or malnutrition is associated with increased postoperative complications, mortality, prolonged hospital stay and higher costs [4,5]. Different from most preoperative risk factors of postoperative complications that cannot be corrected, nutritional risk can be potentially improved by adequate nutritional support. Early detection of nutritional risk would allow for early intervention which could prevent later complications. There is convincing evidence that preoperative improvement of the patient’s nutritional status and early postoperative nutritional support significantly decrease postoperative complications [1,6].Therefore, evaluation of nutritional risk may be helpful to identify patients who are likely to develop postoperative complications and need specific nutritional support.

To date, a number of nutritional screening and assessment tools have been used to assess nutritional risk [7,8]. However, there is still no consensus on which should be used as the best screening tool to detect nutritional risk in surgical patients. The Nutritional Risk Screening 2002 (NRS 2002), documented by a retrospective analysis of 128 randomized controlled trials of nutritional supports, is a reliable, easily applied and reproducible tool for identifying patients at nutritional risk [9]. According to the NRS 2002, nutritional risk is evaluated by three components: nutritional status, severity of disease and patient age. Compared with other screening tools, NRS2002 is based on three variables-weight loss, BMI and amount of food intake in the preceding week in addition to the patients’ age and the severity of the underlying disease. Patients are classified as being at nutritional risk (score 3 or more) or not (score less than 3) according to the total score obtained [9]. Therefore, NRS 2002, which was designed to include measures of both current potential undernutrition and disease severity, is recognized as a more reliable preoperative nutritional screening score compared with the traditional tools. Importantly, the NRS 2002 appears to have higher sensitivity and specificity for predicting complications than other nutritional assessment tools, such as Malnutrition Universal Screening Tool, the Mini Nutritional Assessment or the Nutritional Risk Index [7].

Recently, the NRS 2002 has been applied increasingly in patients who underwent abdominal surgery for nutritional risk assessment and there are various studies examining the correlation of this test and postoperative outcomes. However, the usefulness of the NRS 2002 for predicting is controversial [10–12]. This controversy might due to different kinds of disease, different types of surgery or different population characteristics (i.e. the patients’ age is an important variable to distinguish patients at nutritional risk from patients at non-nutritional risk) in different studies. To our knowledge, no meta-analysis of such studies has been performed on the association between nutritional risk and postoperative outcomes. The aim of this meta-analysis was to examine whether a preoperative evaluation of nutritional risk by NRS 2002 provided prediction of postoperative outcomes in patients undergoing abdominal surgery.

## Methods

### 2.1 Literature search

This meta-analysis was performed according to the PRISMA (Preferred Reporting Items for Systematic Reviews and Meta-analyses) statement (S1 Table). A systematic literature search for published papers was conducted using the following online databases: MEDLINE, EMBASE, the Cochrane library, EBSCO, CRD databases, Cinahl, PsycInfo and BIOSIS previews. The following search terms were used: “nutritional risk”, “nutritional risk score”, “nutritional risk screening 2002”, “NRS”, “NRS 2002”, “abdominal”, “surgery”, “resection”, “operation”, combined with Boolean operators as appropriate. Studies were limited to those published after 2002, as the NRS 2002 was not introduced by the ESPEN until 2002. No language restrictions were applied to the search, and translation was obtained as necessary. We also searched the reference lists of relevant studies for additional studies. For unpublished papers, we searched the ISI Web of Knowledge Conference Proceedings and also included when possible.

### 2.2 Study selection

Studies were included in the meta-analysis if they met the following inclusion criteria: (1) Prospective or retrospective cohort study design; (2) All patients scheduled for surgery at abdominal sites (including gastrointestinal, urological, gynaecological surgery); (3) Pre-operative nutrition risk as determined by the NRS 2002 was recorded; (4) The studies compared the total complication rate, mortality or length of hospital stay (LOS) between patients undergoing abdominal surgery who had pre-operative nutrition risk and those who had not pre-operative nutrition risk; The complications were divided into infective and non-infective complications for the purposes of data analysis. Infective complications included wound infections, abdominal abscess and pneumonia. Non-infective complications included anastomotic leak, wound dehiscence and organ failure. All studies included should have sufficient information to calculate the odds ratio (OR) and 95% CI. We excluded studies that did not provide any of the outcomes mentioned above. Studies that included data of the same population for more than one publication were only included once. Reviews, editorials and case-reports were also excluded.

### 2.3 Data extraction and synthesis

Two investigators independently screened titles and abstracts of all relevant articles according to predetermined inclusion criteria and extracted the data from each included study. The data with the most fully adjusted OR with 95% CI and mean, standard deviation (SD) were extracted from all included studies. If ORs were not available, the data of four-fold table were extracted. If possible, the corresponding authors were contacted for the required data. In addition, we also abstracted the following data from each included study when available: the first author’s name, year of publication, country of origin, number of patients, patient demographics (age, gender), the type of surgery performed, and statistical adjustments for confounding factors. Any disagreements of inclusion, exclusion or data extraction were resolved by consensus or discussion with a third reviewer.

We analyzed the data using the software Review Manager (version 5.2.3 for Windows Copenhagen: The Nordic Cochrane Centre, The Cochrane Collaboration, 2012). For each study, log-OR and the corresponding SE were calculated from the available OR and 95% CI. Pooled ORs and 95% CI were calculated using inverse variance method to evaluate the risk of postoperative complications and mortality. The continuous variable (length of hospital stay) was analysed using a weighted mean difference (WMD). Statistical heterogeneity was assessed using the Cochrane Q test and the I^2^ statistic. P<0.10 or I^2^>50% was considered statistically for heterogeneity [13,14]. If there was significant heterogeneity among studies, a random effects model was used to calculate the most conservative summary estimates; otherwise, a fix effects model was used. Sensitivity analyses were applied to examine the influence of each study on the pooled estimates by omitting every one study and pooling the remaining studies. A funnel plot was constructed to check for evidence of publication bias. p<0.05 was considered statistically significant.

### 2.4 Quality assessment

The methodological quality of all articles included was assessed by the same independent investigators using a checklist based on the Newcastle-Ottawa Scale (NOS) [15]. The checklist was designed for observational research of interventions and consists of three key domains: 1) the Selection of cohorts; 2) the Comparability of cohorts; 3) assessment of Outcome. A study can be awarded a maximum of one star for each numbered item within the Selection and Outcome domains. A maximum of two stars can be given for Comparability domain. Studies with a score of 7 or greater were considered to be of high quality.

## Results

### 3.1 Literature search

**Fig 1:**
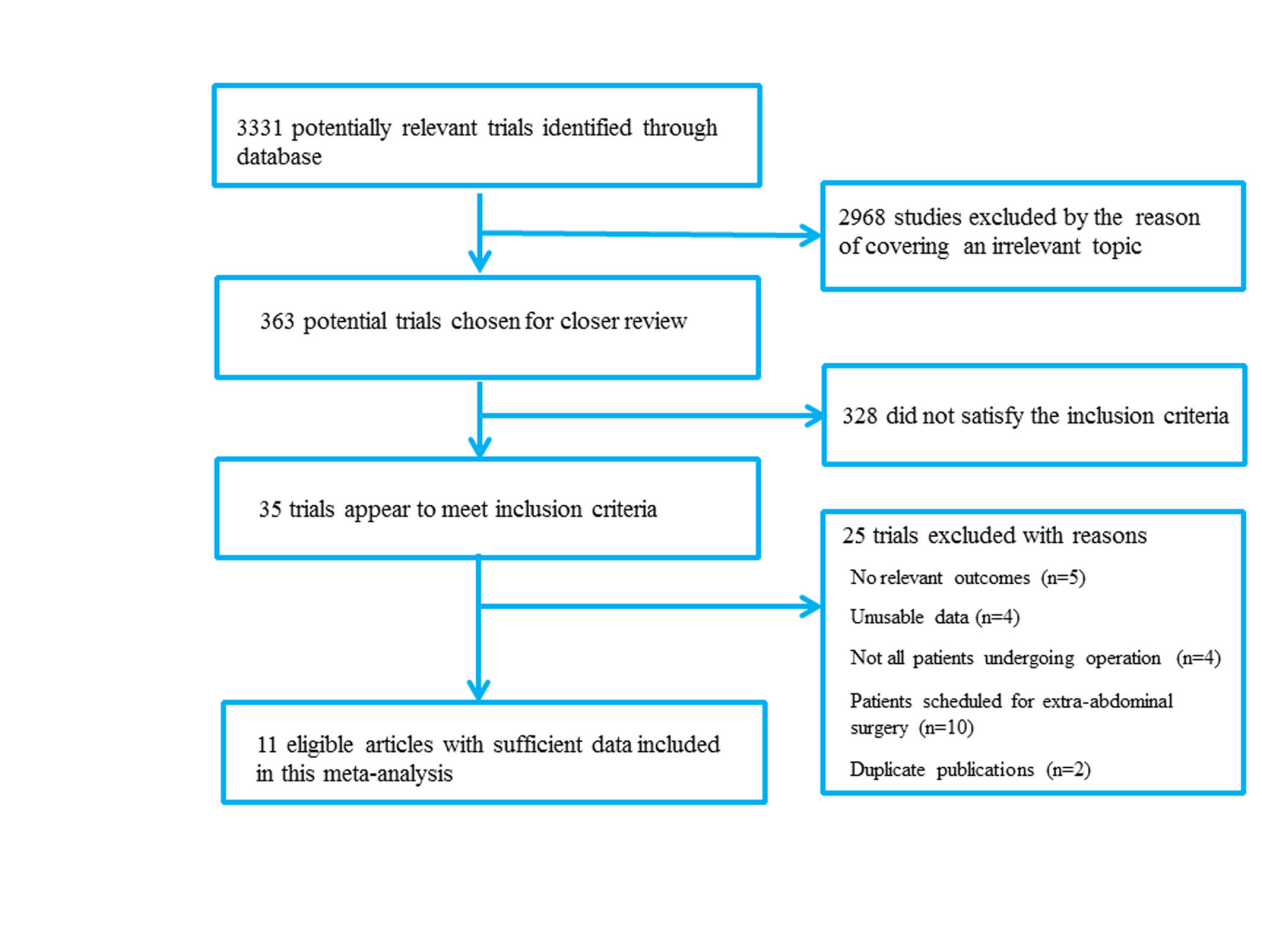
Flow diagram of the study selection process.

There were a total of 3331 potentially relevant studies identified after the application of search strategy. 2968 were excluded by the reason of covering an irrelevant topic after screening the titles and abstracts. Full text of the remaining 363 studies was reviewed and a further 328 studies were excluded for not conforming to the inclusion criterion. Then 25 were excluded for various reasons in detail. Finally, 11 studies [10–12,16–22] met our inclusion criteria and were included in this meta-analysis. Results of the literature search were summarized in Fig 1.

### 3.2 Studies characteristics and quality assessment

Characteristics of the included 11 studies in this meta-analysis are presented in Table 1. The pooled patients included a total of 3527 (range 64–653) patients undergoing abdominal surgery in Asia (7 studies) and Europe (4 studies). All studies were published between 2008 and 2014. The percentage of women included in the meta-analysis ranged from 26% to 47% and the mean age of all patients ranged from about 51 to 66. All studies included patients undergoing abdominal operations, and six studies recruited patients diagnosed with gastric or colorectal cancer and scheduled for an abdominal operation. Most of studies included patients undergoing open surgery, except for one study including patients who were to undergo elective laparoscopic abdominal operation. Seven studies performed preoperative nutritional risk assessment using the NRS 2002 alone, whereas the other four using at least two nutritional assessments. Most of studies adjusted their results for multiple potential demographic and clinical confounders, except for 2 studies, in which such data were not available.

**Table 1:** Patient characteristics of participants in studies included in the meta-analysis.

Study	Country	Diagnosis of patients	Type of surgery	Study design	No. of subjects	Mean age	Female %	Adjustment
**Marc Schiesser 2008**	Switzerland	gastro-intestinal surgery	open surgery	cohort	No risk 521	51.2 (18.0–89.8)	47%	Age, sex, malignant risk, severity of disease
					Risk 87			
**Weiping Guo 2009**	China	gastric carcinoma	open surgery	cohort	No risk 189	NA	NA	NA
					Risk 125			
**I. Schwegler 2010**	Switzerland	colorectal cancer	open surgery	cohort	No risk 113	66.8±12.1	34.90%	Alcohol abuse, smoking, sex, age,
					Risk 73			tumor stage and operative risk
**Wu Li min 2011**	China	colorectal cancer	open surgery	cohort	No risk 184	62.4±12.9	33.20%	Age, preoperative complications
					Risk 105			recent weight loss, surgical history
**D. Kuppinger 2012**	Germany	diseases of the digestive tract or with other abdominal disease	open surgery	cohort	No risk 507	63 (52–69)	42%	presence of edema, ASA grade, duration of operation and number of transfused red cell units
					Risk 146			
**Wei Zhou 2013**	China	NA	laparoscopic operations	cohort	No risk 49	55.83±14.59	41.30%	weight, height, age, sex, albumin values, and ASA grade
					Risk 26			
**Hiroji Shinkawa 2013**	Japan	pancreaticoduodenectomy	open surgery	cohort	No risk 20	65.9 (21–82)	40.60%	NRI, preoperative biliary drainage
					Risk 44			
**Han Dong 2013**	China	gastrointestinal cancer	open surgery	cohort	No risk 137	NA	NA	Age, BMI, albumin values, tumor site and preoperative nutritional support
					Risk 98			
**Liu Hong 2013**	China	rectal cancer	open surgery	cohort	No risk 382	NA	NA	Age, TNM grade
					Risk 259			
**Cerantola 2013**	Switzerland	disease of urinary tract	open surgery	cohort	No risk 59	63±14	24%	Age, gender, BMI, history of smoking or abdominal surgery, ASA score, anemia,
					Risk 51			albumin, Charlson comorbidity index
**Seung-Jin Kwag 2014**	Korea	colorectal cancer	open surgery	cohort	No risk 253	62.9	NA	NA
					Risk 99			

NA, not available; ASA, American Society of Anesthesiologists; NRI, nutritional risk index; BMI, body mass index; TNM, tumor node metastasis.

The quality of studies included in our meta-analysis was high with a mean score of 8, as assessed by the NOS. All studies achieved seven or more stars and were classified as high-quality (Table 2).

**Table 2:** Quality assessment of included studies in the meta-analysis.

Study	1.Representat-iveness of the exposed cohort	2. Selection of the non exposed cohort	3. Ascertainment of exposure	4. Outcome of interest was not present at start of study	5 a. study controls for the most important factor	5 b. study controls for any additional factor	6.Ascertain-ment of outcome	7.Was follow-up long enough for outcomes to occur	8.Adequacy of follow up of cohorts	Total
**Marc Schiesser 2008**	yes	yes	yes	yes	yes	no	no	yes	yes	7
**Weiping Guo 2009**	yes	yes	yes	yes	yes	yes	yes	yes	yes	9
**I. Schwegler 2010**	yes	yes	yes	yes	yes	no	no	yes	yes	8
**Wu Li min 2011**	yes	yes	yes	yes	yes	no	yes	yes	yes	8
**D. Kuppinger 2012**	yes	yes	yes	yes	yes	no	no	yes	yes	7
**Wei Zhou 2013**	yes	yes	yes	yes	yes	yes	yes	yes	yes	9
**Hiroji Shinkawa 2013**	yes	yes	yes	yes	yes	yes	yes	yes	yes	9
**Han Dong 2013**	yes	yes	yes	yes	yes	no	no	yes	yes	7
**Liu Hong 2013**	yes	yes	yes	yes	yes	yes	yes	yes	yes	9
**Cerantola 2013**	yes	yes	yes	yes	yes	no	no	yes	yes	9
**Seung-Jin Kwag 2014**	yes	yes	yes	yes	yes	no	no	yes	yes	7

### 3.3 Association of nutritional risk with post-operative outcomes

#### 3.3.1 Complications

**Fig 2:**
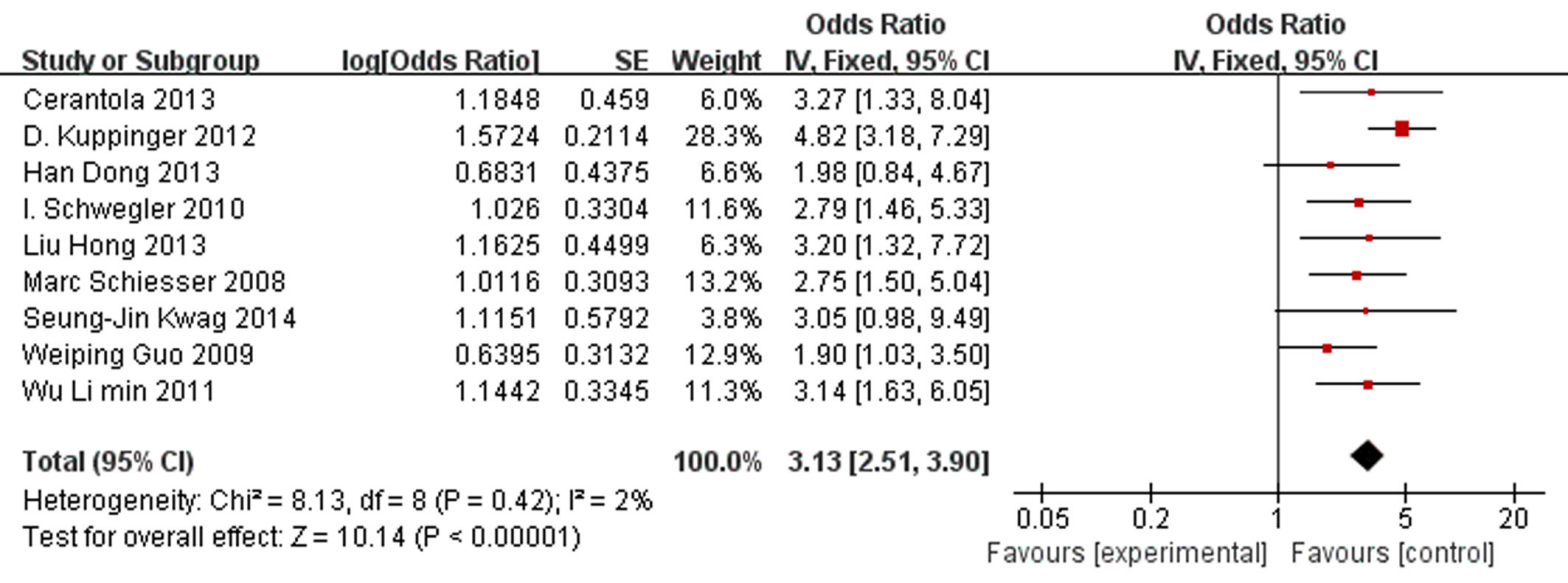
Forest plot showing the effects of nutritional risk group compared to nutritional normal group on overall complications. SE, standard error; IV, inverse variance; CI, confidence interval.

**Fig 3:**
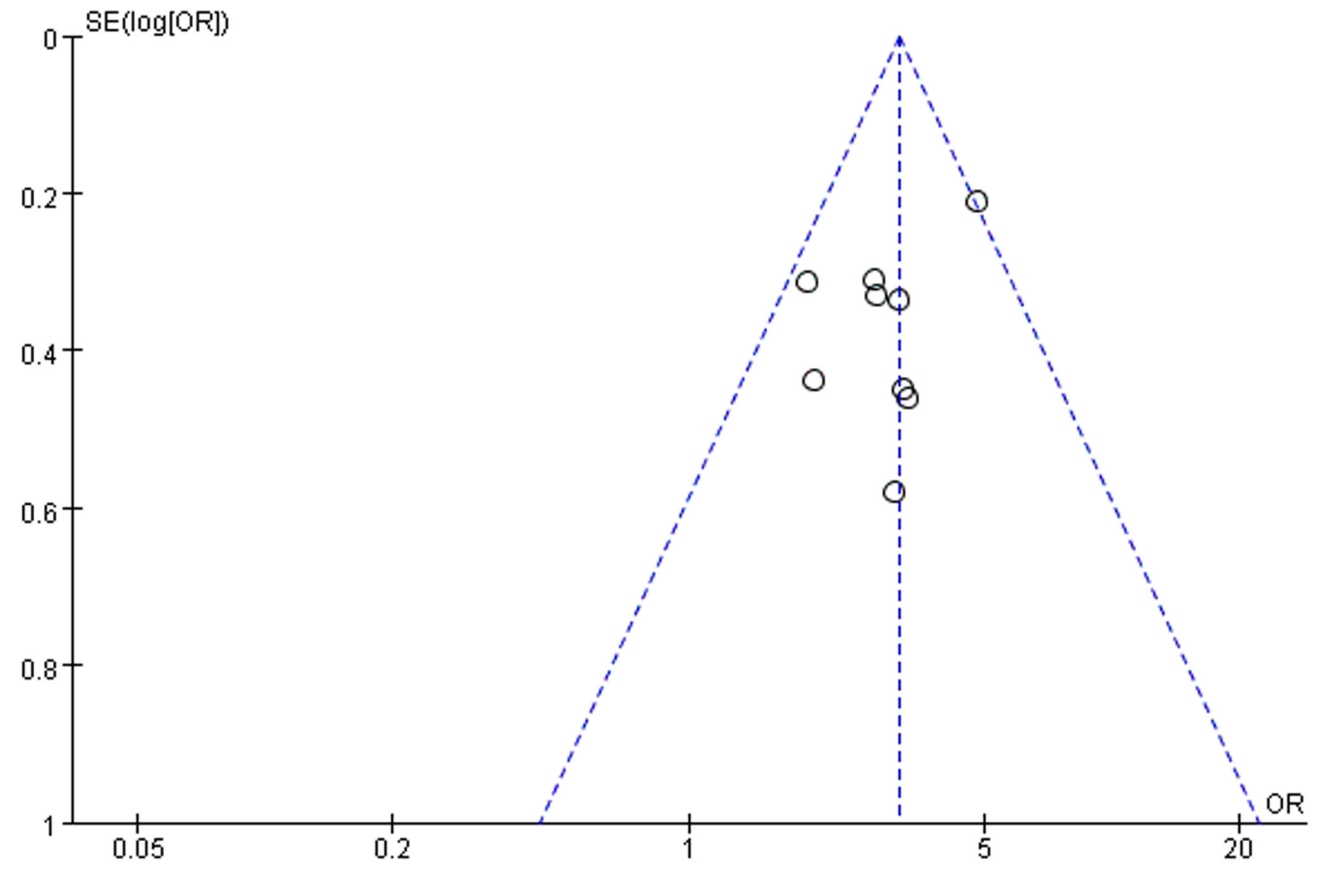
Funnel plots for the overall complications in nutritional risk group compared to nutritional normal group. SE, standard error; OR, odds ratio.

Nine studies reported overall complications in patients undergoing abdominal surgery, with seven studies finding that nutritional risk was a significant predictor of postoperative complications. Fig 2 presented the pooled analysed results of the relationship between nutritional risk defined by NRS 2002 and overall complications. Significant difference was observed in overall complications between patients with nutritional risk and those without. Postoperative overall complications were more frequent in nutritional risk patients versus patients without nutritional risk (the pooled OR 3.13 [2.51, 3.90] p<0.00001). There was no statistical heterogeneity among studies (P = 0.42, I^2^ = 2%). Visual inspection of the funnel plot (Fig 3) suggested asymmetrical distribution of studies, indicating publication bias.

**Fig 4:**
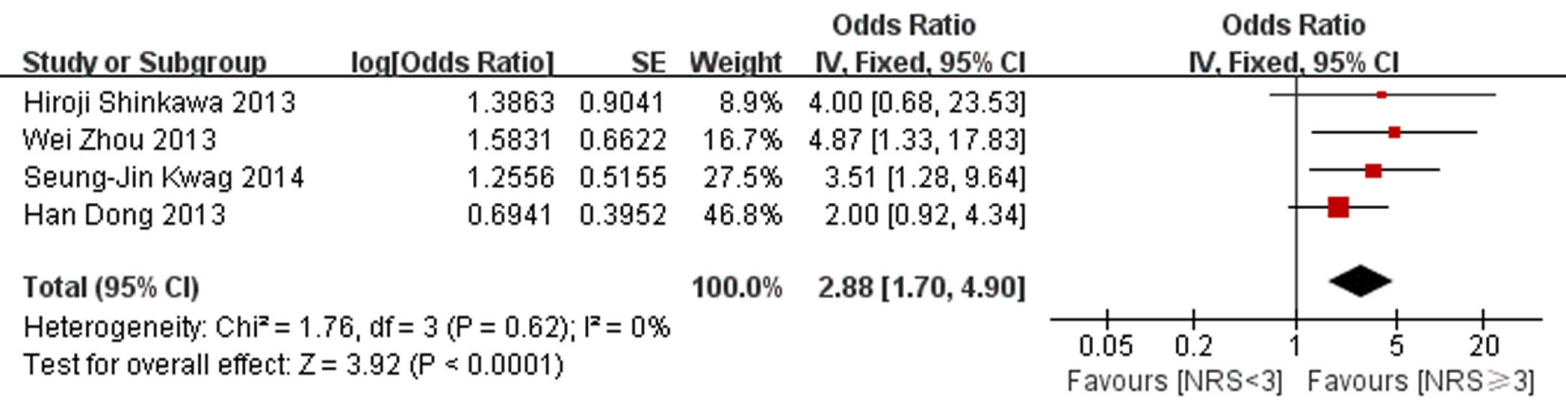
Forest plot showing the effects of nutritional risk group compared to nutritional normal group on infective complications. SE, standard error; IV, inverse variance; CI, confidence interval.

In addition, four studies reported outcomes on infectious complications. As shown in Fig 4, nutritional risk was also significantly associated with an increased risk of infectious complications (the pooled OR 2.88 [1.7, 4.9] p<0.0001). No statistical heterogeneity was found among the studies (P = 0.62, I^2^ = 0%).

#### 3.3.2 Mortality

**Fig 5:**
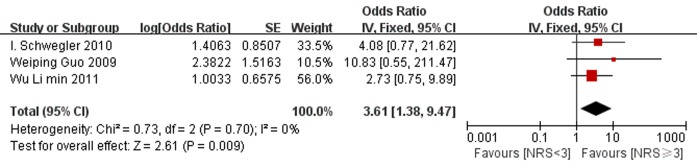
Forest plot showing the effects of nutritional risk group compared to nutritional normal group on mortality. SE, standard error; IV, inverse variance; CI, confidence interval.

Three studies provided the information of mortality in patients with nutritional risk and those without risk. All of these three studies found no significant association between nutritional risk and mortality. However, the pooled OR of mortality for the two groups was 3.61 [1.38, 9.47] (p = 0.009), which meant that patients with nutritional risk had higher mortality rates when compared with patients with no risk. There was no evidence for between-study heterogeneity (P = 0.7, I^2^ = 0%). (Fig 5)

#### 3.3.3 Length of hospital stay

**Fig 6:**
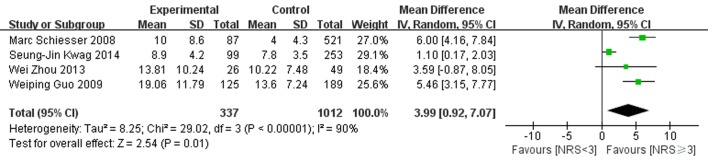
Forest plot showing the effects of nutritional risk group compared to nutritional normal group on length of hospital stay. SE, standard error; IV, inverse variance; CI, confidence interval.

Length of hospital stay was reported in four of the included studies, with two studies finding an association between nutritional risk and length of hospital stay. As presented in Fig 6, the postoperative hospital stay was significant longer in the preoperative nutritional risk group than in the nutritional normal group (WMD 5.58 [4.21, 6.95] p<0.00001). However, there was strong heterogeneity among the studies (P<0.00001, I^2^ = 90%). The sensitivity analysis revealed that there was no significant change in the pooled WMD or 95% CI on excluding any of the studies (WMD lied between 3.22 and 5.58). The study of Senug-Jin Kwag seemed to slightly influence the results, whereas the results did not change materially after exclusion of this study (WMD 5.58 [4.21, 6.95]). In addition, the heterogeneity was removed after leaving out this study (P = 0.61, I^2^ = 0%).

## Discussion

It is widely assumed that preoperative nutritional status is an important determinant of postoperative outcomes in patients following major abdominal surgery. Various aspects of nutritional deficiencies could lead to malnutrition. However, malnutrition is still poorly defined. Unlike the traditional definition of malnutrition, nutrition risk was defined as “chances of a better or worse outcome from disease or surgery, according to actual or potential nutritional and metabolic status.” [23] Nutritional risk is considered potentially reversible through nutritional support treatment, thus early recognition and a validated nutritional risk scoring system may play an important role in improving postoperative outcomes.

According to our meta-analysis, pre-operative nutritional risk was strongly correlated with increased overall and infective complications rates, increased mortality and prolonged hospital stay. Overall, risk of bias was low and quality of included studies was high, suggesting that main results of our study were unequivocal. Surprisingly, low heterogeneity was detected in most analyses, suggesting that most included studies pointed in the same direction, with no major outliers. To some extent, the low heterogeneity made our results more precision. Of course, we cannot rule out that true levels of heterogeneity in our meta-analysis cannot be detected with currently available statistical methods. It was worth noting that there was a significant heterogeneity when combining these studies with regard to the length of hospital stay. The study of Senug-Jin Kwag was exactly identified as the main contributor to heterogeneity through the sensitivity analysis. However, the postoperative LOS was still significant longer in patients with nutritional risk after excluding this study, which suggested that the overall results of our analysis were statistically robust.

Similar to the present findings, other studies also confirmed that nutritional risk was a significant risk factor for postoperative outcomes in various patients. Ozkalkanli et al. found that NRS 2002 could predict development of complications in patients undergoing orthopedic surgery [24]. Tevik K and his colleagues also reported that NRS 2002 was a reliable predictive tool in hospitalized patients with heart failure [25].These findings also leaded to the assumption that patients who are detected as undernutrition risk by NRS 2002, applying a nutritional support both before and after the operation might decrease mortality and the risk of complication, and shorten the LOS.

Various parameters of nutritional assessments had been used to evaluate the nutritional conditions of patients, whereas the most reliable tool for nutritional risk screening had not been clarified [7]. MNA was developed as the most valuable instrument for nutritional assessment of geriatric patients in their homes. SGA and MUST tended to be more subjective to grade malnutrition. NRS2002 had been increasingly used for nutritional assessment recently and it might be an excellent tool, as it was the first one in the world focused on evidence-based medicine. Compared to other tools, NRS 2002 allowed for the gradation of disease effect (score 1–3) and would not miss patients who are at nutritional risk because of specific disease. For patients scheduled for abdominal surgery, severity of disease seemed to be a more important index. Moreover, previous study had demonstrated that NRS 2002 appeared to be a screening tool that better predicted hospital-related outcome than MUST and SGA. Another advantage of NRS 2002 was that it was less time consuming and required less examiner training than other tools. Most screening tools are based on variables such as weight loss, food intake, body mass index and underlying diseases, whereas NRS 2002 had an additional age adjustment for patients over 70 years. Therefore, NRS 2002 tended to identify more elderly patients at nutritional risk. In reality, elderly patients were not always at bad nutritional status. Although this might limit the prediction of NRS 2002 in some cases, NRS 2002 appeared to be still an appropriate tool for evaluation of nutritional status for patients scheduled for abdominal surgery.

The following limitations of the present meta-analysis should be mentioned. First, primary diagnoses of our included studies varied broadly, especially a large portion of patients had malignant disease. Thus, the type of underlying disease would play a significant role in patient’s outcomes. Second, the sample size was relatively small, and some additional studies were still warranted to determine whether our results were still significant in larger populations. Third, most of our included studies lacked detailed information of postoperative management, which could also possibly influence the incidence of postoperative outcomes. Moreover, all studies included in this meta-analysis were involving Asia and Europe population, thus more studies from other races were needed to evaluate the correlation between nutritional risk and post-operative outcomes.

In conclusion, we reinforced the value of nutritional risk as a predictor of postoperative outcomes in patients undergoing abdominal surgery. ESPEN guidelines recommended that the surgical treatment of patients at high nutritional risk should be postponed until there was an improvement in their nutritional state [26]. Moreover, nutritional support was not applicable to all patients on the basis of Kondrup’s relatively efficient philosophy on nutritional support [9]. Therefore, it was necessary for clinical physicians to evaluate objectively the nutritional status of patients’ condition before providing moderate interventions, which may help patients survive the perioperative period. From the present analysis, we expected that NRS 2002 would be helpful in detecting patients at risk of developing nutrition related postoperative outcomes. However, NRS 2002 needs to be validated in larger samples of patients undergoing abdominal surgery by better reference method, to investigate how well NRS 2002 can identify the patients’ nutritional status.

## Supporting Information

S1 Table PRISMA 2009 Checklist. (DOC)
